# ASTER: accurately estimating the number of cell types in single-cell chromatin accessibility data

**DOI:** 10.1093/bioinformatics/btac842

**Published:** 2022-12-27

**Authors:** Shengquan Chen, Rongxiang Wang, Wenxin Long, Rui Jiang

**Affiliations:** School of Mathematical Sciences and LPMC, Nankai University, Tianjin 300071, China; Ministry of Education Key Laboratory of Bioinformatics, Research Department of Bioinformatics at the Beijing National Research Center for Information Science and Technology, Center for Synthetic and Systems Biology, Department of Automation, Tsinghua University, Beijing 100084, China; School of Mathematical Sciences and LPMC, Nankai University, Tianjin 300071, China; Ministry of Education Key Laboratory of Bioinformatics, Research Department of Bioinformatics at the Beijing National Research Center for Information Science and Technology, Center for Synthetic and Systems Biology, Department of Automation, Tsinghua University, Beijing 100084, China

## Abstract

**Summary:**

Recent innovations in single-cell chromatin accessibility sequencing (scCAS) have revolutionized the characterization of epigenomic heterogeneity. Estimation of the number of cell types is a crucial step for downstream analyses and biological implications. However, efforts to perform estimation specifically for scCAS data are limited. Here, we propose ASTER, an ensemble learning-based tool for accurately estimating the number of cell types in scCAS data. ASTER outperformed baseline methods in systematic evaluation on 27 datasets of various protocols, sizes, numbers of cell types, degrees of cell-type imbalance, cell states and qualities, providing valuable guidance for scCAS data analysis.

**Availability and implementation:**

ASTER along with detailed documentation is freely accessible at https://aster.readthedocs.io/ under the MIT License. It can be seamlessly integrated into existing scCAS analysis workflows. The source code is available at https://github.com/biox-nku/aster.

**Supplementary information:**

Supplementary data are available at *Bioinformatics* online.

## 1 Introduction

Rapid advances in single-cell chromatin accessibility sequencing (scCAS) technologies, such as single-cell assay for transposase-accessible chromatin with sequencing, have enabled the characterization of epigenomic heterogeneity and the interrogation of gene regulation at an unprecedented resolution. A number of embedding and clustering methods have been proposed to identify groups of cells with similar epigenomic patterns in scCAS data (Chen *et al.*, 2019, 2021). However, none of these methods suggests the number of cell types present in the data, which is crucial in clustering analysis and can be critical for downstream analyses of single-cell data (Yu *et al.*, 2022).

Several methods have been proposed specifically for cell-type number estimation in single-cell RNA sequencing (scRNA-seq) data (Supplementary Text S1), and their performance has been benchmarked systematically (Yu *et al.*, 2022). Although almost all the widely-used scCAS data analysis workflows, e.g. Signac (Stuart *et al.*, 2021), ArchR (Granja *et al.*, 2021) and EpiScanpy (Danese *et al.*, 2021), adopted community detection-based techniques to find the best possible grouping, the estimation of the number of cell types in scCAS data is still typically subjective and largely relied on the investigator’s desired clustering resolution and/or prior knowledge (Supplementary Text S2).

To address this need, we propose a Python package named ASTER to **a**ccurately e**st**imate the numb**er** of cell types in scCAS data.

## 2 Materials and methods

**Fig. 1.**
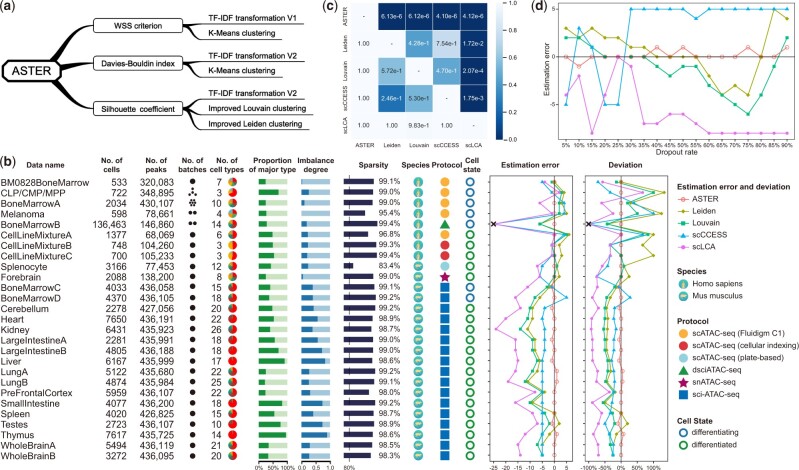
Benchmarking results of various methods based on 27 scCAS datasets. (**a**) The ensemble estimation strategy of ASTER. (**b**) Performance of different methods on datasets generated from different species and protocols, and with various sizes, dimensions, numbers of batches, numbers of cell types, proportions of the major type, degrees of cell-type imbalance, levels of sparsity and cell states. Note that we encountered memory errors (exceeded 256 GB) when performing scCCESS and scLCA on BoneMarrowB. (**c**) *P*-values of one-sided paired Wilcoxon signed-rank tests that test if a method (one of the row names) achieves significantly lower absolute estimation deviation on the 27 datasets than another method (one of the column names). (**d**) The performance of various methods on BoneMarrowA at different dropout rates evaluated by estimation error.

Given a peak-by-cell scCAS data $X\in R^{p\times n}$, ASTER estimates the number of cell types based on ensemble strategies (Fig. 1a). Firstly, ASTER performs estimation based on the within-cluster sum-of-squares (WSS) criterion. Specifically, ASTER applies term frequency-inverse document frequency (TF-IDF) transformation (V1) to matrix X (Supplementary Text S3). ASTER then performs principal component analysis (PCA) using the widely-used EpiScanpy workflow and performs K-Means clustering to measure $\mathrm{WSS}=\sum_{i=1}^{N}\underset{\mu_{j}\in C}{\min}\left({\left\|x_{i}-\mu_{j}\right\|}^{2}\right)$, where N denotes the number of cells, $x_{i}$ the representation of the i-th cell, $\mu_{j}$ the representation of the j-th cluster center and C the resulting clusters. A good clustering is one with a low WSS score and a low number (k) of clusters. However, this is a tradeoff because WSS decreases as k increases. Therefore, we adopt an elbow method to identify the elbow/knee point (the point with maximum curvature) of a k-versus-WSS line (Satopaa *et al.*, 2011). The k of the elbow point is adopted as the optimal number of clusters.

Secondly, ASTER performs estimation based on the Davies–Bouldin index (Davies and Bouldin, 1979). Instead of TF-IDF transformation (V1), ASTER applies another widely-used TF-IDF transformation (V2) to X (Supplementary Text S3). ASTER then performs PCA and K-Means as above to measure the Davies–Bouldin index, which is defined as $\frac{1}{k}\sum_{i=1}^{k}\underset{i\neq j}{\max}\frac{s_{i}+s_{j}}{d_{ij}}$, where k is the number of clusters, $s_{i}$ the average distance between each cell of cluster i and the centroid of that cluster and $d_{ij}$ the distance between cluster centroids i and j. A lower index indicates a better partition, and the k that provides the minimum index is thus adopted as the optimal number of clusters.

Thirdly, ASTER performs estimation based on the silhouette coefficient (Rousseeuw, 1987), which is defined for a single cell as $\frac{b-a}{\max(a,b)}$, where a denotes the mean distance between the cell and all other cells in the same cluster, b denotes the mean distance between the cell and all other cells in the next nearest cluster. ASTER performs TF-IDF transformation (V2) and PCA as above, and then constructs a neighborhood graph of cells using the EpiScanpy workflow. Instead of K-Means clustering, ASTER adopts another two widely-used clustering methods, i.e. Louvain and Leiden, which require a resolution parameter but not the number of clusters. To obtain the desired number of clusters, a binary search strategy is usually adopted (Chen *et al.*, 2019, 2021; Danese *et al.*, 2021). However, each attempt in the search process is time-consuming, especially for large data. To speed up the search process, we further improve the search strategy based on the weighted bias as follows:

$$r_{\mathrm{next}}=r_{\mathrm{this}}+\left(r_{\max}-r_{\min}\right)\times \frac{k-k_{\min}}{k_{\max}-k_{\min}},$$

where $r_{\mathrm{next}}$ and $r_{\mathrm{this}}$ denote the resolutions in the next and this attempt, respectively, $r_{\max}$ and $r_{\min}$ denote the maximum and minimum resolutions to be searched, respectively, $k_{\max}$ and $k_{\min}$ denote the obtained numbers of clusters using the maximum and minimum resolutions, respectively. For each k, ASTER calculates the mean silhouette coefficient of all cells based on Louvain and Leiden clustering results, respectively, and then sums up the two means. A higher silhouette coefficient relates to a model with better-defined clusters, and the k that provides the maximum coefficient is thus adopted as the optimal number of clusters.

Finally, ASTER estimates the number of cell types by averaging the three numbers estimated above, that is, the ensemble estimation is based on three metrics, two TF-IDF approaches, and three clustering methods. Besides, building upon the widely-used AnnData format, ASTER can be seamlessly integrated into the EpiScanpy analysis workflow.

## 3 Results

We evaluated the performance of ASTER by estimation error (the difference between the estimated and true number of cell types) and estimation deviation (the estimation error normalized by the true number of cell types) as recommended in a recent benchmark study (Yu *et al.*, 2022). Note that this task is different from clustering and higher clustering concordance does not necessarily mean a more accurate estimation (Yu *et al.*, 2022). We compared the performance of ASTER with four methods (Supplementary Text S4), including Louvain and Leiden with default resolution, two widely-used methods in scCAS data analysis, and scCCESS and scLCA (Cheng *et al.*, 2019), two of the best methods in the most recent benchmark study for scRNA-seq data (Yu *et al.*, 2022). We collected 27 datasets generated from different protocols, and with various sizes, dimensions, numbers of batches, numbers of cell types, degrees of cell-type imbalance, cell states and levels of sparsity for systematic benchmarking (Supplementary Text S5 and Table S1).

As shown in Figure 1b, ASTER accurately estimates the number of cell types in scCAS data and significantly outperformed the baseline methods. First, ASTER performed well on BM0828BoneMarrow (a dataset of differentiating bone marrow cells from a donor), indicating its ability for datasets where expression changes among cells are expected to be gradients. Second, ASTER performed well on CLP/CMP/MPP (a subset of bone marrow cells from four donors) and BoneMarrowA (the entire dataset of bone marrow cells from seven donors), indicating its ability for datasets derived from multiple batches. However, ASTER does not model the batch variation specifically. Since technical variation may be large in some scCAS datasets, we recommend performing batch correction before estimating the number of cell types by ASTER. Third, ASTER also outperformed other methods on Melanoma (a dataset of cells in time series after knockdown of SOX10 in two short-term patient cultures). Fourth, we evaluated ASTER on BoneMarrowB, a dataset containing 136 463 cells from two batches. ASTER again provided superior performance, indicating its ability for large-scale datasets, which can lead to poor estimation in the benchmark study (Yu *et al.*, 2022). Fifth, in addition to differentiating cell states, we also demonstrated the superior performance of ASTER on three differentiated cell-line mixtures. Sixth, in addition to the above human datasets generated by three various protocols, ASTER also outperformed other methods on 19 mouse datasets generated by another 3 protocols. Seventh, ASTER also performed well on challenging datasets generated from complex tissues and with high degrees of cell-type imbalance. One-sided paired Wilcoxon signed-rank tests further demonstrated that the advantages of ASTER over the baseline methods were significant (Fig. 1c). We provided more details of the above results in Supplementary Text S6 and Figures S1 and S2.

To mimic protocols that generate sparser scCAS data, we downsampled the reads in BoneMarrowA, which provides cell-type labels after fluorescent-activated cell sorting. ASTER consistently outperformed other methods when the dropout rate varied from 5% to 90% (Fig. 1d). We also performed model ablation analysis to demonstrate the advantage of the ensemble strategies of ASTER (Supplementary Text S7 and Figs S3 and S4). Besides, among all the 27 datasets, the improved Louvain and Leiden clustering strategies in ASTER reduced the average number of searches by 4.74 and 2.04, respectively.

## 4 Conclusion

Based on comprehensive experiments on multiple datasets, ASTER provides an accurate way to estimate the number of cell types in scCAS data. We anticipate that ASTER will provide a valuable guidance and greatly assist with refining cell ontology in scCAS data analysis.

## Supplementary Material

btac842_Supplementary_Data
