# Supplementary material for: ASTER: accurately estimating the number of cell types in single-cell chromatin accessibility data
Source: Bioinformatics. 2022 Dec 27;39(1):btac842. doi: 10.1093/bioinformatics/btac842 (PMC9825259; doi:10.1093/bioinformatics/btac842)
Supplement: btac842_Supplementary_Data [file btac842_supplementary_data.pdf]

Supplementary Information

ASTER: accurately estimating the number of cell types in single-cell chromatin accessibility data

Shengquan Chen[1,*], Rongxiang Wang[2], Wenxin Long[1] and Rui Jiang[2,*]

[1]School of Mathematical Sciences and LPMC, Nankai University, Tianjin 300071, China and [2]Ministry of Education Key Laboratory of Bioinformatics, Research Department of Bioinformatics at the Beijing National Research Center for Information Science and Technology, Center for Synthetic and Systems Biology, Department of Automation, Tsinghua University, Beijing 100084, China

* To whom correspondence should be addressed.

Contents

Supplementary Texts 2

Text S1. Ideas of existing methods for scRNA-seq data 2

Text S2. Challenges pertaining to scCAS data 3

Text S3. TF-IDF transformation 4

Text S4. Baseline methods 5

Text S5. Data collection and processing 6

Text S6. Details of the benchmarking results 8

Text S7. Model ablation analysis 13

Supplementary Figures 14

Fig. S1 14

Fig. S2 15

Fig. S3 16

Fig. S4 17

Supplementary Tables 18

Table S1. Summary of the 27 scCAS datasets for benchmarking 18

Table S1 (continue). Summary of the 27 scCAS datasets for benchmarking 19

References 20

Supplementary Texts

Text S1. Ideas of existing methods for scRNA-seq data

To estimate the number of cell types in a given scRNA-seq dataset, a number of remarkable methodologies have been proposed (Yu, et al., 2022). These methodologies can be grouped into four main categories. The first category is based on the idea of community detection, which optimizes community structure to find the best possible grouping and is the most widely-used category in the single-cell data analysis community. The typical methods of this category are Louvain algorithm (Blondel, et al., 2008) and Leiden algorithm (Traag, et al., 2019), which have been adopted in almost all the conventional scRNA-seq data analysis workflows, such as Seurat (Hao, et al., 2021), Scanpy (Wolf, et al., 2018), and Monocle3 (Cao, et al., 2019). We note that Louvain and Leiden also have been adopted in scCAS data analysis workflows, such as Signac (Stuart, et al., 2021), ArchR (Granja, et al., 2021) and EpiScanpy (Danese, et al., 2021). The second category is based on the intuitive idea that a good clustering should have high intra-cluster similarity (cells within a cluster are similar) and low inter-cluster similarity (cells from different clusters are dissimilar). For example, scLCA uses the silhouette coefficient to study the separation distance between the resulting clusters (Cheng, et al., 2019), CIDR adopts the Calinski and Harabasz score to measure the ratio of the sum of inter-cluster dispersion and the sum of intra-cluster dispersion for all clusters (Lin, et al., 2017), and RaceID is based on the Gap statistic that compares the change in intra-cluster dispersion with that expected under an appropriate reference null distribution (Grün, et al., 2015). The third category is based on the idea of eigengap heuristic, which suggests the number of cell types as the value that maximizes the eigengap (difference between consecutive eigenvalues). There are several scRNA-seq data-specific methods belong to this category, such as SIMLR (Wang, et al., 2017), Spectrum (John, et al., 2020) and SC3 (Kiselev, et al., 2017). The fourth category is based on the idea that more stable and reproducible clustering results can be obtained by specifying the optimal number of cell types rather than by specifying a suboptimal number of cell types. The methods of this category are usually incorporated with ensemble learning strategies. For example, scCCESS assumes that clustering using the optimal number of clusters would be the most robust to small perturbations in the scRNA-seq data, and estimates the number of cell types by ensemble clustering (Yu, et al., 2022).

Text S2. Challenges pertaining to scCAS data

Considering that analysis of chromatin accessibility can reveal transcriptional regulatory sequences, many scCAS studies for complex tissues or diseases have been proposed (Buenrostro, et al., 2018; Buenrostro, et al., 2015; Cusanovich, et al., 2015; Cusanovich, et al., 2018; Domcke, et al., 2020; Pijuan-Sala, et al., 2020; Preissl, et al., 2018; Rai, et al., 2020), yielding novel mechanistic and evolutionary insights about the functional landscape of the genome. Compared with scRNA-seq data, scCAS data has assay-specific characteristics such as the close-to-binary nature, extreme sparsity, and tens of times higher dimensions. Although both scRNA-seq data and scCAS data can be expressed in the form of a matrix after preprocessing and the methods designed for scRNA-seq data can be applied to scCAS data theoretically, the characteristics of scCAS data make the application subject to various obstacles. For example, scRNA-seq data-specific methods become much more time-consuming and memory-consuming when they are applied to high-dimension scCAS data, and thus can hardly be applied to large-scale scCAS data from the technical perspective. Therefore, scCAS data analysis has assay-specific challenges and requires a specially designed methodology. Many methods specific to scCAS data analysis have been proposed, such as data embedding and cell clustering methods (Baker, et al., 2019; Bravo Gonzalez-Blas, et al., 2019; Chen, et al., 2021; Cusanovich, et al., 2018; Danese, et al., 2021; Fang, et al., 2021; Granja, et al., 2021; Liu, et al., 2021; Schep, et al., 2017; Xiong, et al., 2019; Zamanighomi, et al., 2018), even though there have been a great many methods for scRNA-seq data analysis. Analogously, although various methods for determining cluster numbers have been benchmarked for scRNA-seq data (Yu, et al., 2022), the estimation of number of cell types in scCAS data requires further methodological innovation.

Text S3. TF-IDF transformation

We apply term frequency-inverse document frequency (TF-IDF) transformation (V1) to peak i and cell j as:

$$x'_{ij} = \frac{x_{ij}}{\sum_{i=1}^{p} x_{ij}} \log\left(\frac{n}{\sum_{j=1}^{n} x_{ij}}\right)$$

which represents how important the peak i is for cell j, and is then normalized as a recent study for scCAS data estimation (Li, et al., 2021):

$$v_{ij} = \frac{x'_{ij}}{\sqrt{\sum_{i=1}^{p} {x'_{ij}}^{2}}}$$

For TF-IDF transformation (V2), we first weight all the peaks in individual cells by the 'term frequency', which is the total number of accessible peaks in that cell, and then multiply the weighted matrix by $\log(1 + \text{inverse document frequency})$, which is the inverse frequency of each peak across all cells (Chen, et al., 2021; Cusanovich, et al., 2018). This step normalizes for sequencing depth, upweights peaks that do not occur very frequently, and downweights prevalent peaks.

Text S4. Baseline methods

Existing methods have mainly focused on the estimation of the number of cell types in scRNA-seq data, whereas efforts to accurately estimate the number of cell types in single-cell chromatin accessibility (scCAS) data are still limited. Here, we compared the performance of ASTER with four baseline methods, including Louvain, Leiden, scCCESS and scLCA. Specifically, Louvain and Leiden are two community detection-based techniques to find the best possible grouping, and have been adopted in almost all the widely-used scCAS data analysis workflows, e.g., Signac (Stuart, et al., 2021), ArchR (Granja, et al., 2021) and EpiScanpy (Danese, et al., 2021). scCCESS is a stability-based approach for estimating the number of cell types by taking advantage of a random sampling-based ensemble deep clustering model (Geddes, et al., 2019; Yu, et al., 2022). scLCA is a machine learning based analytical pipeline that combines similarity measurement by latent cellular states with a graph-based clustering algorithm, and provides heuristic solutions for population number inference (Cheng, et al., 2019). scCCESS and scLCA have provided the overall best performance on the estimation of the number of cell types in scRNA-seq data in the most recent benchmark study (Yu, et al., 2022). Following the standard EpiScanpy analysis workflow for scCAS data, we performed Louvain and Leiden with default resolution to estimate the number of cell types in scCAS data. We performed scCCESS with SIMLR (Wang, et al., 2017) following the tutorial recommended by the benchmarking study (Yu, et al., 2022). We performed scLCA following the tutorial and fed the batch information into the method for the datasets with cells from multiple batches, considering that scLCA provides the option of batch effect correction. The range of optional k ($k > 1$) was set as ten values upstream and downstream of the true number of cell types to be evaluated to find the optimal number. All the benchmark experiments were performed on a machine with two Intel XeonPlatinum 8375C CPUs and 256GB of RAM.

Text S5. Data collection and processing

We collected 27 scCAS datasets generated from different species with 6 different protocols, and with various sizes, dimensions, numbers of batches, numbers of cell types, degrees of cell-type imbalance, cell states, and levels of sparsity for systematic benchmarking.

First, we collected human hematopoietic cells with donor label BM0828 from a bone marrow single-cell ATAC-seq (Fluidigm C1) dataset, and referred the dataset as BM0828BoneMarrow (Buenrostro, et al., 2018). We also collected CLP/CMP/MPP, a subset of bone marrow cells from 4 donors, and BoneMarrowA, the entire dataset of bone marrow cells from 7 donors, from the same study (Buenrostro, et al., 2018). All these three datasets provide cell type labels after fluorescent activated cell sorting. To further test the performance of various methods for differentiating cells, we then collected Melanoma, a dataset of cells in time series after knockdown of SOX10 in melanoma cell lines of two short-term patient cultures (Bravo Gonzalez-Blas, et al., 2019). To assess the performance on large-scale scCAS data, we collected BoneMarrowB, a dataset containing 136,463 cells of 14 differentiating cell types from 2 batches via dsciATAC-seq (Lareau, et al., 2019). In addition to differentiating cell states, we also collected three differentiated human cell-line mixtures (CellLineMixtureA, CellLineMixtureB and CellLineMixtureC) generated by scATAC-seq with Fluidigm C1 or combinatorial cellular indexing to further systematically demonstrate the performance of different methods (Buenrostro, et al., 2015; Cusanovich, et al., 2015).

Second, to investigate if the findings are comparable across different species and data sources, we collected a dataset of mouse splenocytes (referred to as Splenocyte) generated by a plate-based scATAC-seq method after red blood cell removal (Chen, et al., 2018). Besides, we also collected Forebrain, a dataset derived from mouse forebrain by single-nucleus ATAC-seq to test the performance of various methods on cells from complex tissues (Preissl, et al., 2018). Given that most scCAS experiments generate data that capture cell types with different numbers of cells, sometimes with a high degree of cell-type imbalance, we further collected all the 17 datasets in Mouse sci-ATAC-seq Atlas (Cusanovich, et al., 2018) to test the impact of imbalanced ratios of cells among different cell types. The datasets were profiled by a combinatorial indexing assay (sci-ATAC-seq), contain both differentiating and differentiated tissues, and have different levels of sparsity (Cusanovich, et al., 2018).

We used the number of cell types identified in the original studies of the datasets as ground truth for benchmarking. The cell types were identified by experimental fluorescent activated cell sorting (e.g., datasets of BM0828BoneMarrow, CLP/CMP/MPP, and BoneMarrowA), by the various cell lines used to computationally construct the scCAS dataset (e.g., datasets of

CellLineMixtureA, CellLineMixtureB, and CellLineMixtureC), or by unsupervised cell clustering followed by manually assigning the putative cell-type label to each cluster (e.g., datasets of BoneMarrowB, Forebrain, and Heart). The cell-type labels were considered reasonable and have been used to assess unsupervised cell clustering performance and supervised cell type annotation performance (Chen, et al., 2019; Chen, et al., 2021; Chen, et al., 2022; Liu, et al., 2021; Xiong, et al., 2019; Zamanighomi, et al., 2018).

A summary of the collected 27 scCAS datasets for benchmarking is provided in Supplementary Table S1. The imbalance degree of a dataset is defined by estimating the normalized entropy of the cell-type size distribution as follows:

$$I = 1 + \frac{1}{\log C} \sum_{c=1}^{C} \frac{n_c}{N} \log \frac{n_c}{N}$$

where C denotes the number of cell types in the dataset, n_c denotes the number of cells in the c-th cell type, N denotes the total number of cells in the dataset. This index will have value 1 if one cell type has all cells and value 0 if all the cell types have the same number of cells. The sparsity of a dataset is defined as the proportion of zero elements in the scCAS count matrix.

Similar to the existing scCAS data analysis methods (Chen, et al., 2021; Chen, et al., 2022; Liu, et al., 2021; Xiong, et al., 2019; Zamanighomi, et al., 2018), we selected peaks/regions that have at least one read count in at least 1% of the cells in the scCAS count matrix to reduce the noise level. For the downsampling procedure, we randomly dropped out the non-zero entries in the data matrix to zero with a probability equal to the dropout rate as (Chen, et al., 2021; Chen, et al., 2022).

Text S6. Details of the benchmarking results

Analysis of computational time and memory usage

With the increase of scCAS throughput and data scale, the computational approach should meet the basic requirement of efficiency and scalability. We also benchmarked the average computational time and peak memory usage of different methods. Note that both scCCESS and scLCA reported memory errors when running on BoneMarrowB, the largest real scCAS dataset used in this study. We thus subsampled 70% of cells from BoneMarrowB, and failed to perform these two baseline methods again, which indicates that scRNA-seq data-specific methods can hardly be applied to scCAS data, whose dimensions are usually dozens of times higher than that of scRNA-seq data.

We next randomly subsampled 50% cells from BoneMarrowB, repeatedly performed each method 10 times one-by-one on the machine with two Intel XeonPlatinum 8375C CPUs and 256GB of RAM, and recorded the average computational time and peak memory usage. As shown in Supplementary Fig. S1, although fast for computing, scLCA was the most memory-consuming method compared to others and may prohibit its application to large-scale datasets. In contrast, scCCESS required relatively less memory while consuming much more computational time, highlighting a trade-off between memory usage and computational efficiency for the methods. Leiden and Louvain, two similar and straightforward clustering methods, took the least amount of time and had the lowest memory usage. The computational time and memory usage of ASTER were acceptable for conventional personal computers and were moderate among all the methods, which was reasonable since ASTER is based on ensemble strategies. We also evaluated the estimation performance of different methods on this subsampled BoneMarrowB dataset. ASTER accurately estimated the number of cell types in this dataset, while Leiden, Louvain, scCCESS and scLCA had estimation errors of -5, -6, 5 and -9, respectively, and estimation deviations of -35.7%, -42.9%, 35.7% and -64.3%, respectively. Together, the above results suggest that ASTER has satisfactory scalability on the premise of accurate estimation of the number of cell types in scCAS data.

Advancement of ASTER on 27 scCAS datasets

We used 27 datasets generated from different protocols, and with various sizes, dimensions, numbers of batches, numbers of cell types, degrees of cell-type imbalance, cell states, and levels of sparsity for systematic benchmarking. As shown in Fig. 1b, ASTER accurately estimated the number of cell types in 19 of the 27 datasets and had low absolute estimation

deviation on the other 8 datasets, providing superior and robust performance. However, scCCESS and scLCA, two of the state-of-the-art methods specific to scRNA-seq data (Yu, et al., 2022), almost always provided over-estimation (positive deviation) or under-estimation (negative deviation) in the experiments and had poor scalability to the scale of scCAS data. Besides, ASTER had average reductions of 62.5% and 92.1% in estimation deviation compared to scCCESS and scLCA, respectively (Supplementary Fig. S2). We further showed the significance of the advantages of ASTER by conducting one-sided paired Wilcoxon signed-rank tests to test if ASTER achieves significantly lower absolute estimation deviation than another method. As shown in Fig. 1c, the absolute estimation deviation of ASTER was significantly lower than all the baseline methods, indicating that the state-of-the-art methods specific to scRNA-seq data no longer have advantages in scCAS data.

We also benchmarked the performance of the widely-used Louvain and Leiden methods to estimate the number of cell types, following the standard EpiScanpy analysis workflow for scCAS data. Note that these two strategies have been adopted in almost all the widely-used scCAS data analysis workflows, e.g., Signac (Stuart, et al., 2021), ArchR (Granja, et al., 2021) and EpiScanpy (Danese, et al., 2021). Since these two straightforward strategies are based on the results of data processing and dimension reduction for scCAS data, they can be regarded as methods specific to scCAS data. However, as shown in Fig. 1b, these two methods also achieved only limited performance to estimate the number of cell types in scCAS data and the performance fluctuated greatly across different datasets. Again, ASTER provided significantly lower absolute estimation deviation than these two methods (Fig. 1c and Supplementary Fig. S2), indicating that the widely-used methods specific to scCAS data still have a lot of room for improvement.

Given that ASTER is also based on Louvain and Leiden, we next looked deep into the estimation results of ASTER_SC (an individual branch of ASTER which is based on silhouette coefficients), Louvain, and Leiden to explain why ASTER was not influenced by the results from Louvain and Leiden algorithms. Taking the dataset of Kidney as an example, Louvain and Leiden performed worst on this dataset according to the estimation errors (i.e., -14 and -11, respectively), while ASTER_SC provided an estimation error of -5. Although all the three methods under-estimated the number of cell types in this dataset, the estimation result of ASTER_SC was closer to the ground-truth number. Taking the dataset of CLP/CMP/MPP as another example, Louvain and Leiden performed worst on this dataset according to the estimation deviations (i.e., 100.0% and 133.3%, respectively), while ASTER_SC provided an estimation deviation of 33.3%. Although all the three methods over-estimated the number of cell types in this dataset, the estimation result of ASTER_SC was closer to the ground-truth number. The results suggest that ASTER_SC effectively integrates the advantages of Louvain,

Leiden and the silhouette coefficient-based estimation strategy. Besides, we couducted one-sided paired Wilcoxon signed-rank tests to test if the ensemble of two branches in ASTER achieves significantly lower absolute estimation deviation than Louvain and Leiden on the 27 benchmarking datasets. ASTER_DB_SC, which is based on the Davies-Bouldin index and the silhouette coefficient, achieved significantly lower absolute estimation deviation than Louvain and Leiden with P-values of 1.35e-5 and 9.05e-6, respectively. ASTER_WSS_SC, which is based on the WSS criterion and the silhouette coefficient, achieved significantly lower absolute estimation deviation than Louvain and Leiden with P-values of 3.01e-4 and 9.21e-4, respectively. The results suggest that the ensemble strategies makes ASTER robust to the results from Louvain and Leiden algorithms.

Performance on imbalanced scCAS datasets

We have shown the performance of ASTER on datasets with various imbalance degrees (Fig. 1b). Although ASTER only accurately estimated the number of cell types in two (SmallIntestine and LargeIntestineB) of the 5 datasets with top imbalance degrees (Thymus, Liver, SmallIntestine, Testes, and LargeIntestineB with imbalance degrees of 0.902, 0.833, 0.737, 0.737, and 0.725, respectively), ASTER consistently provided lower absolute estimation deviation compared to the four baseline methods, indicating ASTER has superior performance on imbalanced datasets. We looked deep into the estimation results of the branches in ASTER to reveal how ASTER achieves accurate estimation results. Taking the dataset of SmallIntestine as an example, the number of cell types is 18, while the estimation results of the branches in ASTER (i.e., ASTER_WSS, ASTER_DB and ASTER_SC) were 16, 23, and 13, respectively. It is intuitive that an individual branch in ASTER can hardly accurately estimate the number of cell types and has significantly higher absolute estimation deviation than the ensemble-based ASTER method (Supplementary Text S7). In this example, ASTER_WSS and ASTER_SC tended to provide under-estimation, while ASTER_DB tended to provide over-estimation. The ensemble of these branches then led to more accurate estimation, which suggests that the ensemble strategies have the main contribution to the effectiveness of ASTER on imbalanced scCAS datasets.

To mimic datasets with rare cell types, we used simATAC, a scCAS data simulation framework, to simulate cells of various cell types (Navidi, et al., 2021). More specifically, we used the dataset of BM0828BoneMarrow as the input of simATAC. BM0828BoneMarrow contains seven hematopoietic cell types which were identified by fluorescent activated cell sorting and can be regarded as ground truth labels. We simulated 50 CLP cells (common lymphoid

progenitors) and simulated 500 cells for each of the remaining six cell types, resulting in a dataset of 3050 cells with a rare cell type that accounts for only 1.639%. The estimation result of ASTER was 7, while the results of Leiden, Louvain, scCCESS and scLCA were 9, 8, 12 and 7, respectively. The results indicate that ASTER and scLCA accurately estimated the number of cell types, while Leiden, Louvain and scCCESS tended to provide over-estimation when there is only one rare cell type in the scCAS data. We next simulated a dataset of 10 000 cells containing 9700 CLP cells and 50 cells for each of the remaining six cell types. The imbalance degree of this dataset is 0.903 and is higher than that of all the 27 benchmarking datasets. The estimation result of ASTER was 7, while the results of Leiden, Louvain, scCCESS and scLCA were 9, 8, 4 and 2, respectively. The results indicate that ASTER again accurately estimated the number of cell types, Leiden and Louvain again tended to provide over-estimation, while scCCESS and scLCA tended to provide under-estimation when there is only one major cell type in the scCAS data.

Taken together, the above results suggest that the ensemble-based ASTER method can effectively deal with size imbalance of clusters, e.g., from rare cell types, compared to the baseline methods.

Performance on large scCAS datasets

We have benchmarked the estimation performance of different methods on BoneMarrowB, a dataset containing 136,463 cells of 14 differentiating cell types from 2 batches via droplet single-cell assay for transposase-accessible chromatin using sequencing (dsciATAC-seq) (Lareau, et al., 2019). However, scCCESS and scLCA reported memory errors when running on this dataset. To further evaluate the methods on large datasets, we collected 37,818 cells profiled via a massively parallel droplet-based method in primary tumor biopsies from 7 patients with basal cell carcinoma (BCC) (Satpathy, et al., 2019). There are 20 tumor microenvironment (TME) cell types in this dataset. We again encountered memory errors (exceeded 256 GB) when performing scCCESS and scLCA on this dataset. ASTER and Louvain accurately estimated the number of cell types in this dataset, while Leiden provided over-estimation with error of 4. The results again demonstrate the effectiveness and scalability of ASTER to estimate the number of cell types in droplet-based datasets.

To evaluate the methods on much larger datasets, we used simATAC (Navidi, et al., 2021) to simulate cells with ground-truth cell-type labels based on the dataset of BoneMarrowA, which contains 10 hematopoietic cell types. Specifically, we simulated 20,000 cells for each cell type, and obtained a dataset of 200,000 cells. As expected, scCCESS and scLCA failed to work on

this large dataset again. ASTER accurately estimated the number of cell types in this dataset, while both Leiden and Louvain provided over-estimation with error of 7 and deviation of 70%. The results suggest that ASTER can scale well to large datasets.

Performance on high-noise scCAS datasets

To mimic protocols that generate sparser scCAS data, we downsampled the reads in BoneMarrowA. We have looked deep into the performance of Louvain and Leiden when the dropout rate varies from 5% to 90%, and we found an interesting phenomenon that with the dropout rate increases, Louvain and Leiden constantly under-estimated the number of cell types and then over-estimated the number of cell types after reaching an inflection point. Specifically, the number estimated by either Louvain or Leiden had an obvious decreasing trend when the dropout rate varied from 5% to 75%, and had an obvious increasing trend when the dropout rate varied from 75% to 90%. To quantify the decreasing and increasing trends, we adopted the Pearson correlation coefficient to measure the linear relationship between the dropout rate and the estimated number of cell types. When the dropout rate ranged from 5% to 75%, the Pearson correlation coefficients between the dropout rate and the numbers estimated by Louvain, Leiden and ASTER_SC were -0.946, -0.947 and 0.274, respectively, and the P-values of tests (the null hypothesis is the distributions underlying the samples are uncorrelated and normally distributed) were 9.46e-8, 8.76e-8 and 3.23e-1, respectively. When the dropout rate ranged from 75% to 90%, the Pearson correlation coefficients between the dropout rate and the numbers estimated by Louvain, Leiden and ASTER_SC were 0.996, 0.910 and -0.258, respectively, and the P-values of tests (the null hypothesis is the distributions underlying the samples are uncorrelated and normally distributed) were 4.09e-3, 8.97e-2 and 7.42e-1, respectively. Therefore, the reason Louvain and Leiden had lower performance when the dropout rate was low but relatively higher performance when it was high is that either Louvain or Leiden began with over-estimation, and had two chances to achieve more accurate estimation since the estimated number first decreased and then increased when the dropout rate varied from 5% to 90%. Besides, we noticed that ASTER_SC, a branch of ASTER that is based on Louvain and Leiden algorithms, was robust to the dropout rate, which again suggests the advantages of our ensemble strategies.

Text S7. Model ablation analysis

ASTER is based on the branches of the WSS criterion (WSS), the Davies-Bouldin index (DB), and the silhouette coefficient (SC). We also evaluated the performance of the ASTER variants with only a single branch (ASTER_WSS, ASTER_DB and ASTER_SC) or with the ensemble of two branches (ASTER_WSS_DB, ASTER_DB_SC and ASTER_WSS_SC). As shown in Supplementary Fig. S3, ASTER provided obviously lower absolute estimation deviation compared with the variants. ASTER had average reductions of 11.5%, 20.9%, 28.4%, 12.0%, 4.53% and 12.7% in estimation deviation compared to ASTER_WSS, ASTER_DB, ASTER_SC, ASTER_WSS_DB, ASTER_DB_SC and ASTER_WSS_SC, respectively (Supplementary Fig. S4a). One-sided paired Wilcoxon signed-rank tests also demonstrated that the advantages of ASTER over the six variants were significant (Supplementary Fig. S4b). The results indicate that although based on scCAS data-specific processing approaches, the estimation strategies using either individual or a combination of two metrics cannot guarantee satisfactory performance, highlighting the contribution of ensemble strategies in ASTER.

Supplementary Figures

Fig. S1

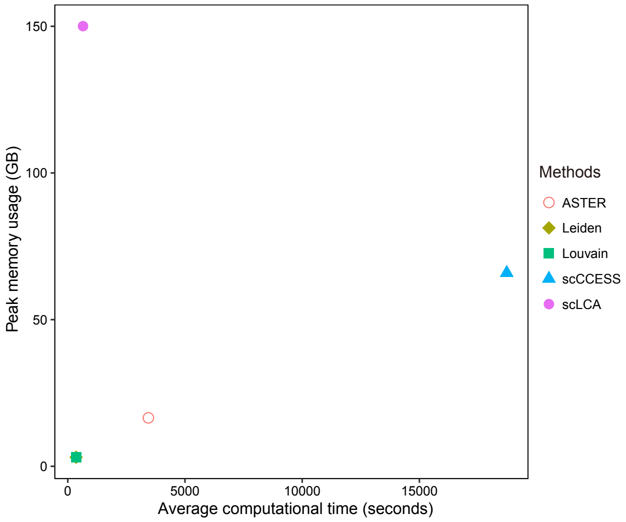

Fig. S1. Average running time and peak memory usage of different methods in 10 repeated experiments on the subsampled 50% of cells in BoneMarrowB.

Fig. S2

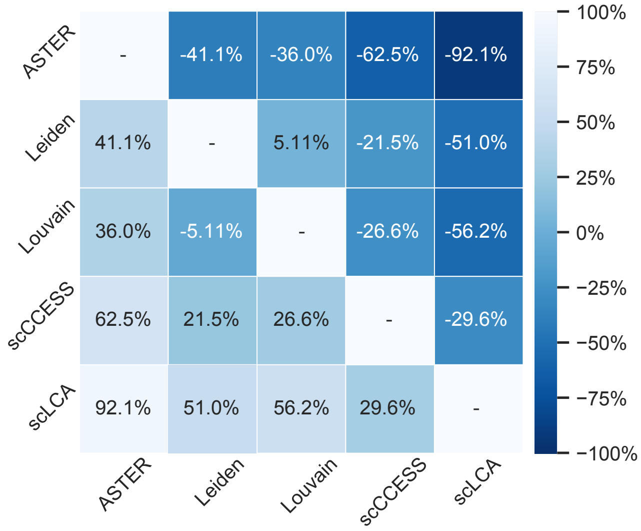

Fig. S2. Average difference of absolute estimation deviation between a method (one of the row names) and another method (one of the column names).

Fig. S3

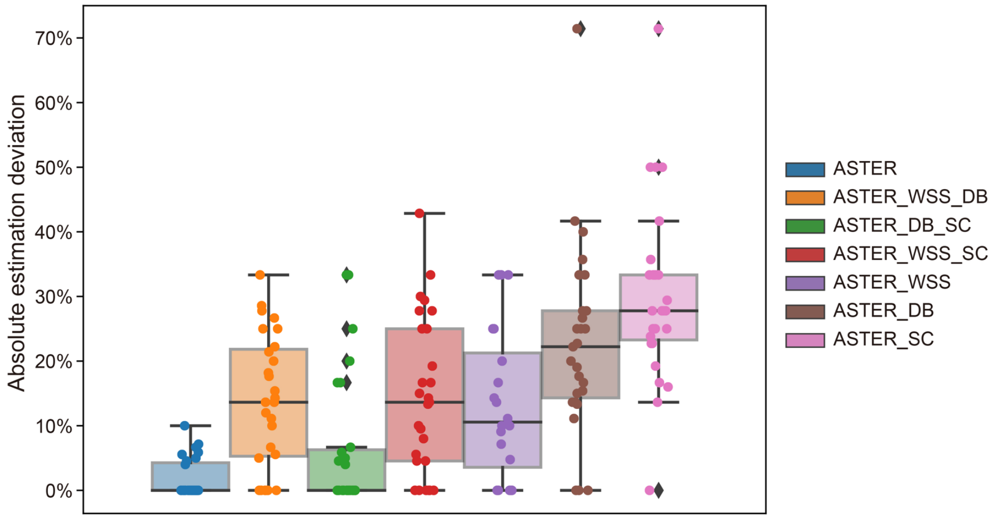

Fig. S3. Absolute estimation deviation of ASTER and its six variants on 27 scCAS datasets.

Fig. S4

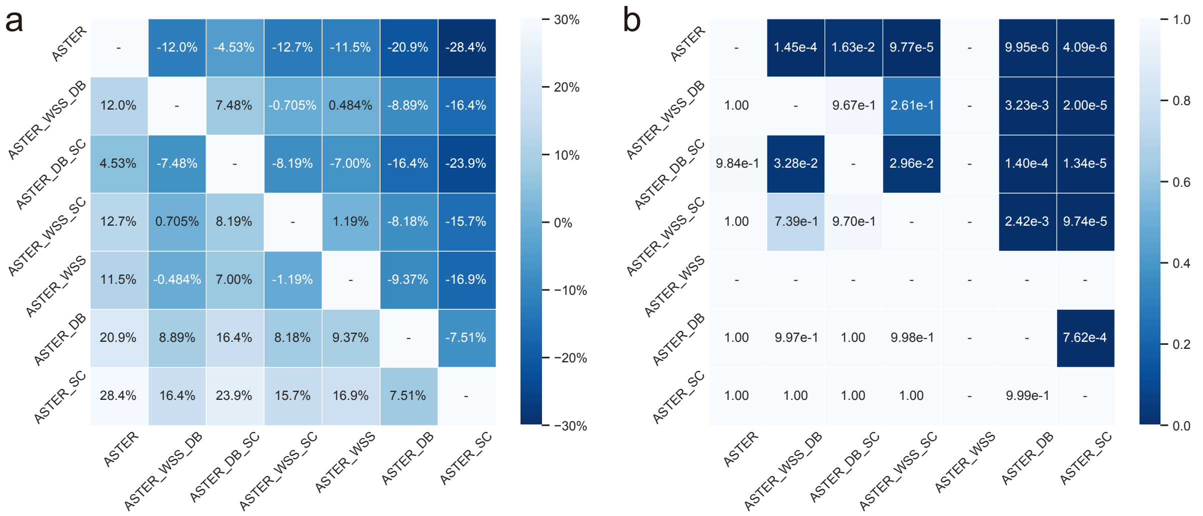

a	ASTER	ASTER_WSS_DB	ASTER_DB_SC	ASTER_WSS_SC	ASTER_WSS	ASTER_DB	ASTER_SC
ASTER	-	-12.0%	-4.53%	-12.7%	-11.5%	-20.9%	-28.4%
ASTER_WSS_DB	12.0%	-	7.48%	-0.705%	0.484%	-8.89%	-16.4%
ASTER_DB_SC	4.53%	-7.48%	-	-8.19%	-7.00%	-16.4%	-23.9%
ASTER_WSS_SC	12.7%	0.705%	8.19%	-	1.19%	-8.18%	-15.7%
ASTER_WSS	11.5%	-0.484%	7.00%	-1.19%	-	-9.37%	-16.9%
ASTER_DB	20.9%	8.89%	16.4%	8.18%	9.37%	-	-7.51%
ASTER_SC	28.4%	16.4%	23.9%	15.7%	16.9%	7.51%	-

b	ASTER	ASTER_WSS_DB	ASTER_DB_SC	ASTER_WSS_SC	ASTER_WSS	ASTER_DB	ASTER_SC
ASTER	-	1.45e-4	1.63e-2	9.77e-5	-	9.95e-6	4.09e-6
ASTER_WSS_DB	1.00	-	9.67e-1	2.61e-1	-	3.23e-3	2.00e-5
ASTER_DB_SC	9.84e-1	3.28e-2	-	2.96e-2	-	1.40e-4	1.34e-5
ASTER_WSS_SC	1.00	7.39e-1	9.70e-1	-	-	2.42e-3	9.74e-5
ASTER_WSS	-	-	-	-	-	-	-
ASTER_DB	1.00	9.97e-1	1.00	9.98e-1	-	-	7.62e-4
ASTER_SC	1.00	1.00	1.00	1.00	-	9.99e-1	-

Fig. S4. Performance comparison of ASTER and its six variants. (a) Average difference of absolute estimation deviation between a method (one of the row names) and another method (one of the column names). (b) P-values of one-sided paired Wilcoxon signed-rank tests that test if a method (one of the row names) achieves significantly lower absolute estimation deviation on the 27 datasets than another method (one of the column names). Note that ASTER_WSS failed to find the elbow point on several datasets.

Supplementary Tables

Table S1. Summary of the 27 scCAS datasets for benchmarking

Data name	No. of cells	No. of peaks	No. of batches	No. of cell types	Imbalance degree	Sparsity	Species	Protocol	Cell state
BM0828BoneMarrow	533	320,083	1	7	0.024	0.991	Homo sapiens	scATAC-seq (Fluidigm C1)	differentiating
CLP/CMP/MPP	722	348,895	4	3	0.260	0.990	Homo sapiens	scATAC-seq (Fluidigm C1)	differentiating
BoneMarrowA	2034	430,107	7	10	0.106	0.990	Homo sapiens	scATAC-seq (Fluidigm C1)	differentiating
Melanoma	598	78,661	2	4	0.001	0.954	Homo sapiens	scATAC-seq (Fluidigm C1)	differentiating
BoneMarrowB	136,463	146,860	2	14	0.186	0.994	Homo sapiens	dsciATAC-seq	differentiating
CellLineMixtureA	1377	68,069	1	6	0.225	0.968	Homo sapiens	scATAC-seq (Fluidigm C1)	differentiated
CellLineMixtureB	748	104,260	1	3	0.230	0.993	Homo sapiens	scATAC-seq (combinatorial cellular indexing)	differentiated
CellLineMixtureC	700	105,233	1	3	0.204	0.994	Homo sapiens	scATAC-seq (combinatorial cellular indexing)	differentiated
Splenocyte	3166	77,453	1	12	0.235	0.834	Mus musculus	scATAC-seq (plate-based)	differentiated
Forebrain	2088	138,200	1	8	0.054	0.990	Mus musculus	snATAC-seq	differentiated
BoneMarrowC	4033	436,058	1	15	0.397	0.991	Mus musculus	sci-ATAC-seq	differentiating
BoneMarrowD	4370	436,105	1	18	0.399	0.992	Mus musculus	sci-ATAC-seq	differentiating
Cerebellum	2278	427,056	1	20	0.427	0.992	Mus musculus	sci-ATAC-seq	differentiated
Heart	7650	436,191	1	22	0.568	0.989	Mus musculus	sci-ATAC-seq	differentiated
Kidney	6431	435,923	1	26	0.389	0.987	Mus musculus	sci-ATAC-seq	differentiated
LargeIntestineA	2281	435,991	1	18	0.572	0.990	Mus musculus	sci-ATAC-seq	differentiated
LargeIntestineB	4805	436,188	1	18	0.725	0.990	Mus musculus	sci-ATAC-seq	differentiated
Liver	6167	435,999	1	17	0.833	0.986	Mus musculus	sci-ATAC-seq	differentiated
LungA	5122	435,680	1	22	0.274	0.992	Mus musculus	sci-ATAC-seq	differentiated

Table S1 (continue). Summary of the 27 scCAS datasets for benchmarking

Data name	No. of cells	No. of peaks	No. of batches	No. of cell types	Imbalance degree	Sparsity	Species	Protocol	Cell state
LungB	4874	435,984	1	25	0.262	0.991	Mus musculus	sci-ATAC-seq	differentiated
PreFrontalCortex	5959	436,107	1	22	0.305	0.980	Mus musculus	sci-ATAC-seq	differentiated
SmallIntestine	4077	436,200	1	18	0.737	0.992	Mus musculus	sci-ATAC-seq	differentiated
Spleen	4020	426,825	1	15	0.416	0.987	Mus musculus	sci-ATAC-seq	differentiated
Testes	2723	436,107	1	10	0.737	0.989	Mus musculus	sci-ATAC-seq	differentiated
Thymus	7617	435,725	1	14	0.902	0.986	Mus musculus	sci-ATAC-seq	differentiated
WholeBrainA	5494	436,119	1	21	0.295	0.985	Mus musculus	sci-ATAC-seq	differentiated
WholeBrainB	3272	436,095	1	20	0.273	0.983	Mus musculus	sci-ATAC-seq	differentiated

References

Baker, S.M., et al. Classifying cells with Scasat, a single-cell ATAC-seq analysis tool. Nucleic acids research 2019;47(2):e10-e10.

Blondel, V.D., et al. Fast unfolding of communities in large networks. Journal of Statistical Mechanics: Theory and Experiment 2008;2008(10).

Bravo Gonzalez-Blas, C., et al. cisTopic: cis-regulatory topic modeling on single-cell ATAC-seq data. Nat. Methods 2019;16(5):397-400.

Buenrostro, J.D., et al. Integrated Single-Cell Analysis Maps the Continuous Regulatory Landscape of Human Hematopoietic Differentiation. Cell 2018;173(6):1535-1548 e1516.

Buenrostro, J.D., et al. Single-cell chromatin accessibility reveals principles of regulatory variation. Nature 2015;523(7561):486-490.

Cao, J., et al. The single-cell transcriptional landscape of mammalian organogenesis. Nature 2019;566(7745):496-502.

Chen, H., et al. Assessment of computational methods for the analysis of single-cell ATAC-seq data. Genome Biol. 2019;20(1):241.

Chen, S., et al. RA3 is a reference-guided approach for epigenetic characterization of single cells. Nat. Commun. 2021;12(1).

Chen, X., et al. Cell type annotation of single-cell chromatin accessibility data via supervised Bayesian embedding. Nat. Mach. Intell. 2022;4(2):116-126.

Chen, X., et al. A rapid and robust method for single cell chromatin accessibility profiling. Nat. Commun. 2018;9(1):5345.

Cheng, C., et al. Latent cellular analysis robustly reveals subtle diversity in large-scale single-cell RNA-seq data. Nucleic Acids Res 2019;47(22):e143.

Cusanovich, D.A., et al. Multiplex single cell profiling of chromatin accessibility by combinatorial cellular indexing. Science 2015;348(6237):910-914.

Cusanovich, D.A., et al. A Single-Cell Atlas of In Vivo Mammalian Chromatin Accessibility. Cell 2018;174(5):1309-1324 e1318.

Danese, A., et al. EpiScanpy: integrated single-cell epigenomic analysis. Nat. Commun. 2021;12(1).

Domcke, S., et al. A human cell atlas of fetal chromatin accessibility. Science 2020;370(6518).

Fang, R., et al. Comprehensive analysis of single cell ATAC-seq data with SnapATAC. Nat Commun 2021;12(1):1337.

Geddes, T.A., et al. Autoencoder-based cluster ensembles for single-cell RNA-seq data analysis. BMC Bioinformatics 2019;20(Suppl 19):660.

Granja, J.M., et al. ArchR is a scalable software package for integrative single-cell chromatin accessibility analysis. Nat. Genet. 2021;53(3):403-411.

Grün, D., et al. Single-cell messenger RNA sequencing reveals rare intestinal cell types. Nature 2015;525(7568):251-255.

Hao, Y., et al. Integrated analysis of multimodal single-cell data. Cell 2021;184(13):3573-3587.e3529.

John, C.R., et al. Spectrum: Fast density-aware spectral clustering for single and multi-omic data. Bioinformatics 2020;36(4):1159-1166.

Kiselev, V.Y., et al. SC3: Consensus clustering of single-cell RNA-seq data. Nature Methods 2017;14(5):483-486.

Lareau, C.A., et al. Droplet-based combinatorial indexing for massive-scale single-cell chromatin accessibility. Nat. Biotechnol. 2019;37(8):916-924.

Li, Z., et al. Chromatin-accessibility estimation from single-cell ATAC-seq data with scOpen. Nat. Commun. 2021;12(1):6386.

Lin, P., Troup, M. and Ho, J.W.K. CIDR: Ultrafast and accurate clustering through imputation for single-cell RNA-seq data. Genome Biology 2017;18(1).

Liu, Q., et al. Simultaneous deep generative modeling and clustering of single cell genomic data. Nat. Mach. Intell. 2021;3(6):536-544.

Navidi, Z., Zhang, L. and Wang, B. simATAC: a single-cell ATAC-seq simulation framework. Genome Biology 2021;22(1).

Pijuan-Sala, B., et al. Single-cell chromatin accessibility maps reveal regulatory programs driving early mouse organogenesis. Nat Cell Biol 2020;22(4):487-497.

Preissl, S., et al. Single-nucleus analysis of accessible chromatin in developing mouse forebrain reveals cell-type-specific transcriptional regulation. Nat. Neurosci. 2018;21(3):432-439.

Rai, V., et al. Single-cell ATAC-Seq in human pancreatic islets and deep learning upscaling of rare cells reveals cell-specific type 2 diabetes regulatory signatures. Mol Metab 2020;32:109-121.

Satpathy, A.T., et al. Massively parallel single-cell chromatin landscapes of human immune cell development and intratumoral T cell exhaustion. Nat Biotechnol 2019;37(8):925-936.

Schep, A.N., et al. chromVAR: inferring transcription-factor-associated accessibility from single-cell epigenomic data. Nature methods 2017;14(10):975-978.

Stuart, T., et al. Single-cell chromatin state analysis with Signac. Nat. Methods 2021;18(11):1333-1341.

Traag, V.A., Waltman, L. and van Eck, N.J. From Louvain to Leiden: guaranteeing well-connected communities. Sci. Rep. 2019;9(1).

Wang, B., et al. Visualization and analysis of single-cell RNA-seq data by kernel-based similarity learning. Nat. Methods 2017;14(4):414-416.

Wolf, F.A., Angerer, P. and Theis, F.J. SCANPY: large-scale single-cell gene expression data analysis. Genome biology 2018;19(1):15.

Xiong, L., et al. SCALE method for single-cell ATAC-seq analysis via latent feature extraction. Nat. Commun. 2019;10(1):4576.

Yu, L., et al. Benchmarking clustering algorithms on estimating the number of cell types from single-cell RNA-sequencing data. Genome Biol. 2022;23(1).

Zamanighomi, M., et al. Unsupervised clustering and epigenetic classification of single cells. Nat. Commun. 2018;9(1):2410.
